# A Retrospective Study of Clinical Biomarkers of Severe Dengue in a Tertiary Hospital in Johor Bahru, Malaysia

**DOI:** 10.3390/tropicalmed10020030

**Published:** 2025-01-21

**Authors:** Si Yin Kok, Ruth Elizabeth Abraham, Shareen Nisha Jauhar Ali, Wei Xuan Tuang, Edmund Liang Chai Ong

**Affiliations:** 1 Department of Medicine, Newcastle University Medicine Malaysia, Johor Bahru 79200, Johor, Malaysia; kok.siyin@gmail.com (S.Y.K.); ruthabraham4999@gmail.com (R.E.A.); shareennisha.jauharali@nhs.net (S.N.J.A.); 2 Hospital Sultanah Aminah, Johor Bahru 80100, Johor, Malaysia; wxq321@hotmail.com

**Keywords:** dengue virus, severe dengue, clinical biomarkers, ICU, non-ICU

## Abstract

Management of severe dengue remains a clinical challenge. This retrospective study evaluated clinical features and laboratory biomarkers associated with severe dengue at Hospital Sultanah Aminah Johor Bahru from 1 January 2022 to 31 March 2023. Records of 99 patients, categorized into ICU (51) and non-ICU (48) groups, were identified and analyzed using SPSS version 28.0. Sociodemographic details, clinical features and laboratory biomarkers were collected. Patients aged 50 and older, those with obesity, and those with pre-existing comorbidities were significantly more likely to be admitted to the ICU. The four most common warning signs in both cohorts were lethargy/restlessness/confusion, abdominal pain, persistent vomiting, and diarrhea. Fever, or history of fever, and thrombocytopenia were the two most common severe dengue criteria present in both cohorts. ICU patients exhibited more signs of plasma leakage and abnormal laboratory findings, including normal white cell count, hypoalbuminemia, hyperbilirubinemia, and elevated creatine kinase. In contrast, leukopenia and normal albumin, bilirubin, and creatine kinase levels were more common in non-ICU patients. Hyponatremia and raised lactate dehydrogenase were seen in both groups. This study highlighted key differences and similarities in clinical features and laboratory biomarkers between ICU and non-ICU patients, emphasizing the need for further research to develop a comprehensive risk assessment tool for predicting severe dengue that resulted in ICU admission.

## 1. Introduction

Dengue fever is endemic in over 100 countries and remains the most common communicable disease in Malaysia, with an incidence rate of 397.71 per 100,000 individuals. Johor Bahru has one of the highest rates, with cases increasing from 4400 in 2018 to 9412 in 2020 [1]. Factors such as urbanization, increased global travel, and lack of focused public health measures have contributed to this increasing prevalence. The COVID-19 pandemic further exacerbated dengue cases due to lockdowns and movement control orders, which delayed diagnosis and treatment, disrupted surveillance, and strained healthcare systems [1].

Warning signs of dengue include mucosal bleeding, abdominal pain, and hepatomegaly, potentially requiring intensive care. Severe dengue is defined as plasma leakage causing shock, severe bleeding, respiratory distress or organ dysfunction. While clinical features aid early detection, identifying reliable laboratory biomarkers is essential to identify high-risk individuals for timely and effective management. This study evaluated key clinical features and laboratory biomarkers associated with severe dengue in patients admitted to Hospital Sultanah Aminah Johor Bahru (HSAJB), a tertiary hospital in Malaysia.

## 2. Materials and Methods

This retrospective study, conducted between 1 January 2022 and 31 March 2023, investigated the clinical features and laboratory markers associated with severe dengue in hospitalized adult patients. Patients were identified through hospital census records and categorized into two groups: ICU patients and non-ICU patients. Inclusion criteria required patients to be Malaysian citizens aged 12 years or older who were admitted to Hospital Sultanah Aminah (HSA) for the management or investigation of severe dengue. Patients aged 12 years or younger, those without a confirmed diagnosis of severe dengue or those discharged before completing required investigations were excluded.

Dengue was diagnosed in patients based on the Clinical Practice Guidelines (CPG) and the WHO classification, which categorizes dengue into probable dengue, dengue with warning signs, and severe dengue. Laboratory confirmation was achieved using dengue antigen (NS1 antigen) and serology tests, ensuring accurate case identification. Once severe dengue was confirmed, patient records were analyzed throughout their inpatient stay to assess and analyzed the presence of clinical features, warning signs, severe dengue criteria, and laboratory markers. Clinical features that were identified and analyzed on initial presentation included fever, nausea, vomiting, rash, aches, and pains, while warning signs such as abdominal pain, persistent vomiting, and mucosal bleeding were also included. Severe dengue was identified in patients with severe plasma leakage, significant bleeding, or severe organ involvement, such as liver dysfunction or CNS symptoms. Laboratory findings included increased hematocrit, reduced platelet counts, hypoalbuminemia and hyponatremia, among others.

Data collection was performed using a structured Google Form with sections for sociodemographic data (age, gender, ethnicity), health status (weight, height, BMI, pre-existing conditions, medication history), clinical features, warning signs, severe dengue criteria, laboratory biomarkers and treatment settings (ICU vs. non-ICU). The study initially reviewed the records of 200 patients with suspected severe dengue. However, 95 records were missing and 16 were incomplete, resulting in the analysis of 99 confirmed cases of severe dengue.

The study adhered to the ethical principles outlined in the Declaration of Helsinki (1964) and the Malaysian Good Clinical Practice Guideline. Ethical approval was obtained from the Medical Research and Ethics Committee, Ministry of Health Malaysia (NMRR ID-23-00690-KTK (IIR)), and the University of Newcastle upon Tyne, UK (31879/2023). Data analysis was conducted using SPSS Version 28.0, with categorical variables summarized using frequencies and percentages and continuous variables presented as means or medians.

## 3. Results

In total, 99 patients with suspected severe dengue were included in the analysis, with 51 patients in the ICU cohort and 48 in the non-ICU cohort. Patients aged 50 and above were significantly more likely to require ICU admission. BMI was categorized based on the WHO standards for Asian people; non-obese people (≤27.4) and obese people (≥27.5) [2,3]. Obese individuals were significantly more likely to be admitted to the ICU. The presence of comorbidities such as diabetes mellitus, hypertension and dyslipidemia significantly increased the risk of ICU admission (Table 1).

The three most common clinical features in both ICU and non-ICU cohorts were fever, nausea and vomiting, and arthralgia. The four most common warning signs in both cohorts were lethargy, restlessness or confusion, abdominal pain, persistent vomiting and diarrhea. The two most common severe dengue criteria in both cohorts were fever, or a history of fever within the previous 2 to 7 days and thrombocytopenia. Plasma leakage, including pleural effusion, ascites, and hypoproteinemia, was the third most common sign observed in ICU patients but was less frequent among non-ICU patients. Instead, bleeding from mucosa and hypotension were more commonly seen in non-ICU patients (Table 2).

In both cohorts, a considerable proportion had normal hemoglobin (Hb) levels and normal hematocrit (Hct) levels. More ICU patients had normal white cell counts (WCCs), while more non-ICU patients had leukopenia. Both cohorts predominantly had thrombocytopenia and elevated lactate dehydrogenase (LDH). Hyponatremia, elevated creatinine kinase, elevated bilirubin levels and hypoalbuminemia were more common in the ICU cohort. Among ICU patients, most had normal prothrombin time (PT), elevated activated partial thromboplastin time (APTT), and normal fibrinogen levels. However, coagulation screens were not routinely performed for non-ICU patients, limiting further analysis (Table 3).

## 4. Discussion

Our study showed a significant association between advanced age and the risk of ICU admission, with individuals aged 50 and above being five times more likely to require intensive care. The gradual decline in immune function in the elderly may explain this observation. Obesity was significantly associated with severe dengue, leading to a higher likelihood of requiring intensive care among obese individuals. Similar findings were found in studies by Gallagher et al. [4]. and Chen et al. [5]. Proposed mechanisms include suppression of AMP–protein kinase (AMPK) activity, increased pro-inflammatory adipokines and reactive oxygen species, as well as suppressed function of NK cells, B cells and T cells; all of these mechanisms promote viral replication and tissue damage [4]. Our study observed that pre-existing medical comorbidities significantly increased the risk of ICU admission, with a threefold higher likelihood, aligning with findings by Lien et al. [6] and Ng et al. [7].

The predominant clinical features in both cohorts were fever, nausea and vomiting and arthralgia, whilst the most common warning signs were lethargy/restlessness/confusion, abdominal pain, and persistent vomiting. The most frequent severe dengue criteria in our study included fever, thrombocytopenia, plasma leakage, mucosal bleed and hypotension. Our findings are comparable to other studies. A meta-analysis by Yuan et al. [8] concluded that persistent vomiting, plasma leakage, hepatomegaly, and bleeding significantly increase the risk of severe dengue. Similarly, a meta-analysis by Htun et al. [9] identified vomiting and abdominal pain as important warning signs, followed by bleeding, plasma leakage, elevated hematocrit with reduced platelet counts, hepatomegaly and elevated ALT and AST levels.

Our study reported that over half of the patients in both groups had normal hematocrit levels, with only a small proportion showing elevated levels. Only a small subset (8 out of 99) had hemoconcentration. These suggested that elevated Hct levels or hemoconcentration may not always reliably indicate severe dengue. This observation is supported by Isa et al., which reported normal median Hct levels in ICU patients with severe dengue [10]. Leukopenia was more common among non-ICU patients, whereas ICU patients predominantly had normal WCC levels. This discrepancy may be due to the critical condition of ICU patients and greater physiological stress, promoting leukocytosis [10]. Thrombocytopenia was predominant in both cohorts, aligning with our current understanding. Hyponatremia was also common in both cohorts, with a higher frequency observed among ICU patients. Only a limited number of studies have explored hyponatremia in dengue fever [11], and more research is needed to fully understand the significance of hyponatremia in the progression of dengue.

Elevated CK levels were more common among ICU patients. Additionally, there were relatively few confirmed cases of myositis, but a significant number of patients reported myalgia and arthralgia along with elevated CK levels. This may suggest that muscle involvement is more prevalent than previously recognized with milder symptoms. Many patients in both cohorts had elevated LDH levels, which aligns with findings from previous studies [12,13]. Elevated ALT and AST levels were seen in many patients from both cohorts, consistent with previous studies [12,13]. However, our study reported that a higher proportion of ICU patients had elevated bilirubin, which may suggest that elevated bilirubin was more pronounced in severe cases [14,15]. Similarly, hypoalbuminemia was more common in ICU patients. LDH may predict development of severe dengue, while bilirubin may predict liver failure in patients with dengue. Hypoalbuminemia may be an important prognostic indicator in dengue, irrespective of the day of illness; this is supported by Huy et al. [16].

The routine use of coagulation screens in all severe dengue patients remains a subject of debate. In our hospital, coagulation screens are typically performed in cases of severe dengue with ICU admissions or when hemorrhagic signs are present. Adane et al. observed prolonged prothrombin time (PT) and activated partial thromboplastin time (APTT) in a significant proportion of dengue patients, which is consistent with our findings [17]. Further research is needed to assess the prognostic value of APTT and its application in clinical practice.

## 5. Strengths and Limitations

Our study involved a comprehensive analysis of the demographics, clinical features, warning signs, and laboratory biomarkers associated with severe dengue, highlighting key clinical features and laboratory markers more commonly associated with severe dengue. The goal was to assist clinicians in the prompt identification of severe dengue cases in routine practice, thereby enhancing treatment strategies. A primary limitation of the study was the incomplete documentation of medical records, which limited the sample size, hence limiting the generalizability to other populations. Non-uniformity in data availability due to the missing data in non-ICU patients may have skewed some comparisons.

## 6. Conclusions

In conclusion, this study highlighted the importance of investigating patients presenting with fever, persistent vomiting, diarrhea, abdominal pain, and lethargy for dengue fever. However, these symptoms alone are not specific to dengue. Specifically, the presence of thrombocytopenia, elevated levels of CK, LDH, AST, ALT, bilirubin and hypoalbuminemia should raise concern regarding potential progression to severe dengue and the potential need for ICU admission. Further research is needed to determine the prognostic value of these laboratory markers and their role in predicting ICU admissions and patient outcomes in severe dengue, with the aim of developing a comprehensive validated scoring system incorporating both laboratory markers and clinical features.

## Figures and Tables

**Table 1:** Sociodemographic data.

	ICU (n = 51) n (%)	Mean (Median)	Non-ICU (n = 48) n (%)	Mean (Median)	OR (95% CI)	*p*-Value
A. Sociodemographics						
Gender						0.07
Male	29 (56.9)		18 (37.5)		2.20 (0.98–4.92)	
Female	22 (43.1)		30 (62.5)		0.46 (0.20–1.02)	
Age		42.02 (38.00)		30.46 (28.00)		0.0011
<50	31 (60.8)		43 (89.6)		0.18 (0.061–0.53)	
≥50	20 (39.2)		5 (10.4)		5.55 (1.88–16.39)	
Ethnicity						
Malay	30 (58.8)		31 (64.6)			
Chinese	16 (31.4)		9 (18.8)			
Indian	2 (2.9)		4 (8.3)			
Other	3 (5.9)		4 (8.3)			
Weight		71.04 (66.0)		62.55 (60.00)		
Height		161.07 (162.00)		160.02 (160.00)		
BMI		27.06 (26.00)		24.22 (24.70)		0.04
≤27.4	25 (49.0)		35 (72.9)		0.35 (0.14–0.89)	
≥27.5	20 (39.2)		10 (20.8)		2.8 (1.12–7.00)	
Missing	6 (11.8)		3 (6.3)			
Medical Comorbidities						0.0081
Present	29 (56.9)		14 (29.2)		3.2 (1.39–7.37)	
Absent	22 (43.1)		34 (70.8)		0.31(0.14–0.72)	

Legend: The prevalence, odds ratio (OR), and *p*-value for various sociodemographic characteristics including gender, age, ethnicity, BMI and presence or absence of medical comorbidities with comparison between patients admitted to ICU and non-ICU. The data illustrated the distribution and statistical significance of each characteristic in the study population. *p*-values indicate the level of statistical significance, with *p* < 0.05 considered significant.

**Table 2:** Clinical features, warning signs, and severe dengue criteria.

	ICU (n = 51) n (%)	Non-ICU (n = 48) n (%)
B. Clinical Features		
Fever		
Present	51 (100.0)	48 (100.0)
Absent	0 (0.0)	0 (0.0)
Nausea/Vomiting		
Present	35 (68.6)	36 (75.0)
Absent	16 (31.4)	12 (25.0)
Arthralgia		
Present	31 (60.8)	27 (56.3)
Absent	20 (39.2)	21 (43.8)
Rash		
Present	7 (13.7)	9 (18.8)
Absent	44 (86.3)	39 (81.3)
Leukopenia		
Present	16 (31.4)	18 (37.5)
Absent	35 (68.6)	30 (62.5)
Others		
Present	23 (45.1)	20 (41.7)
Absent	28 (54.9)	28 (58.3)
C. Warning Signs		
Lethargy, restless, confusion		
Present	43 (84.3)	38 (79.2)
Absent	8 (15.7)	10 (20.8)
Abdominal Pain		
Present	25 (49.0)	17 (35.4)
Absent	26 (51.0)	31 (64.6)
Persistent Vomiting ≥ 3 times (over last 24 h)		
Present	24 (47.1)	26 (54.2)
Absent	27 (52.9)	22 (45.8)
Persistent Diarrhea ≥ 3 times (over last 24 h)		
Present	23 (45.1)	23 (47.9)
Absent	28 (54.9)	25 (52.1)
Clinical Fluid Accumulation		
Present	20 (39.2)	5 (10.4)
Absent	31 (60.8)	43 (89.6)
Mucosal Bleed		
Present	3 (5.9)	11 (22.9)
Absent	48 (94.1)	37 (77.1)
Liver enlargement ≥ 2 cm		
Present	7 (13.7)	3 (6.3)
Absent	43 (84.3)	45 (93.8)
Increase in Hct concurrent with rapid decrease in platelet count		
Present	20 (39.2)	15 (31.3)
Absent	31 (60.8)	33 (68.8)
Others		
Present	2 (4.0)	2 (4.0)
Absent	49 (96.1)	46 (95.8)
Severe Dengue Criteria		
Fever or history of fever (2–7 days)		
Present	51 (100.0)	48 (100.0)
Absent	0 (0.0)	0 (0.0)
Thrombocytopenia (100,000 cells per mm3 or less)		
Present	44 (86.3)	30 (62.5)
Absent	7 (13.7)	18 (37.5)
Signs of plasma leakage: pleural effusion, ascites and hypoproteinemia		
Present	30 (58.8)	10 (20.8)
Absent	21 (41.2)	38 (79.2)
Petechiae, ecchymoses or purpura		
Present	8 (15.7)	5 (10.4)
Absent	43 (84.3)	43 (89.6)
Bleeding from mucosa, gastrointestinal tract, injection sites or other locations		
Present	7 (13.7)	13 (27.1)
Absent	44 (86.3)	35 (72.9)
Hematemesis or malena		
Present	2 (3.9)	1 (2.1)
Absent	49 (96.1)	47 (97.9)
Rise in Hct ≥ 20% above average for age/sex		
Present	8 (15.7)	1 (2.1)
Absent	43 (84.3)	47 (97.9)
Drop in Hct following volume replacement treatment ≥ 20% of baseline		
Present	5 (9.8)	3 (6.3)
Absent	46 (90.2)	45 (93.8)
Rapid and weak pulse		
Present	5 (9.8)	6 (12.5)
Absent	46 (90.2)	42 (87.5)
Narrow pulse pressure < 20 mmHg		
Present	4 (7.8)	5 (10.4)
Absent	47 (92.2)	43 (89.6)
Hypotension for age (SBP <90 or SBP drop > 40 from baseline)		
Present	6 (11.8)	12 (25.0)
Absent	45 (88.2)	36 (75.0)
Cold clammy skin and restlessness		
Present	3 (5.9)	2 (4.2)
Absent	48 (94.1)	46 (95.8)
Elevated liver enzymes: AST or ALT ≥ 1000		
Present	11 (21.6)	3 (6.3)
Absent	40 (78.4)	45 (93.8)
CNS: impaired consciousness or reduced GCS		
Present	2 (3.9)	1 (2.1)
Absent	49 (96.1)	47 (97.9)
Myocarditis		
Present	5 (9.8)	0 (0.0)
Absent	46 (90.2)	48 (100.0)
Other		
Present	16 (31.4)	15 (31.4)
Other	35 (68.6)	33 (68.8)

Legend: Comparison between the percentage of patients exhibiting various clinical features, warning signs and severe dengue symptoms between those admitted to the ICU and those in non-ICU wards. The data highlighted the differences in the prevalence of these characteristics between the two groups.

**Table 3:** Laboratory markers.

	ICU (n = 51) n (%)	Non-ICU (n = 48) n (%)
Hb		
Low (<120)	7 (13.7)	13 (27.1)
Normal (120–150)	25 (49.0)	22 (45.8)
Raised (>150)	15 (29.4)	12 (25.0)
Missing	4 (7.8)	1 (2.1)
Hct		
Low (<0.36)	7 (13.7)	11 (22.9)
Normal (0.36–0.46)	29 (56.9)	28 (58.3)
Raised (>0.46)	11 (21.6)	9 (18.8)
Missing	4 (7.8)	0 (0.0)
Total White Cell Count		
Low (<4)	17 (33.3)	28 (58.3)
Normal (4–10)	26 (51.0)	19 (39.6)
Raised (>10)	4 (7.8)	1 (2.1)
Missing	4 (7.8)	0 (0.0)
Platelet		
Low (<150)	46 (90.2)	38 (79.2)
Normal (150–410)	1 (2.0)	10 (20.8)
Missing	4 (7.8)	0 (0.0)
Creatinine		
Low (<44)	5 (9.8)	4 (8.3)
Normal (44–80)	23 (45.1)	26 (54.2)
Raised (>80)	19 (37.3)	9 (18.8)
Missing	4 (7.8)	9 (18.8)
Urea		
Low (<2.8)	11 (21.6)	16 (33.3)
Normal (2.8–8.1)	23 (45.1)	23 (47.9)
Raised (>8.1)	11 (21.6)	0 (0.0)
Missing	6 (11.8)	9 (18.8)
Sodium		
Low (<136)	36 (70.6)	22 (45.8)
Normal (136–145)	10 (19.6)	18 (37.5)
Raised (>145)	1 (2.0)	8 (16.7)
Missing	4 (7.8)	0 (0.0)
Creatine Kinase		
Low (<26)	1 (2.0)	0 (0.0)
Normal (26–132)	13 (25.5)	19 (39.6)
Raised (132)	33 (64.7)	14 (29.2)
Missing	4 (7.8)	15 (31.3)
LDH		
Normal (135–214)	2 (3.9)	1 (2.1)
Raised (>214)	45 (88.2)	35 (72.9)
Missing	4 (7.8)	12 (25.0)
Total Bilirubin		
Normal (<21)	12 (23.5)	36 (75.0)
Raised (>21)	35 (68.6)	5 (10.4)
Missing	4 (7.8)	7 (14.6)
ALT		
Normal (5–33)	2 (3.9)	16 (33.3)
Raised (>33)	45 (88.2)	26 (54.2)
Missing	4 (7.8)	6 (12.5)
AST		
Normal (5–32)	3 (5.9)	7 (14.6)
Raised (>32)	44 (86.3)	33 (68.8)
Missing	4 (7.8)	8 (16.7)
ALP		
Low (<35)	1 (2.0)	2 (4.2)
Normal (35–104)	31 (60.8)	35 (72.9)
High (>104)	15 (29.4)	5 (10.4)
Missing	4 (7.8)	6 (12.5)
Total protein		
Low (<66)	27 (52.9)	11 (22.9)
Normal (66–87)	20 (39.2)	27 (56.3)
Missing	4 (7.8)	10 (20.8)
Albumin		
Low (<35)	35 (68.6)	18 (37.5)
Normal (35–52)	12 (23.5)	23 (47.9)
Missing	4 (7.8)	7 (14.6)
Globulin		
Low (<20)	2 (3.9)	1 (2.1)
Normal (20–35)	32 (62.7)	31 (64.6)
Raised (>35)	13 (25.5)	9 (18.8)
Missing	4 (7.8)	7 (14.6)
Albumin-Globulin Ratio		
Low (<1)	0 (0.0)	11 (22.9)
Normal (1–2)	48 (94.1)	30 (62.5)
Missing	3 (5.9)	7 (14.6)
PT		
Normal (9.5–12)	32 (62.7)	8 (16.7)
Raised (>12)	15 (29.4)	2 (4.2)
Missing	4 (7.8)	38 (79.2)
APTT		
Normal (25.3–37.4)	10 (19.6)	3 (6.3)
Raised (>37.4)	36 (70.6)	7 (14.6)
Missing	5 (9.8)	38 (79.2)
Fibrinogen		
Low (<2)	3 (5.9)	1 (2.1)
Normal (2–4)	42 (82.4)	7 (14.6)
Raised (>4)	2 (3.9)	0 (0.0)
Missing	4 (7.8)	40 (83.3)
NS1		
Positive	32 (62.7)	34 (70.8)
Negative	5 (9.8)	2 (4.2)
Missing	14 (27.5)	12 (25.0)
IgG		
Positive	31 (60.8)	15 (31.3)
Negative	14 (27.5)	23 (47.9)
Missing	6 (11.8)	10 (20.8)
IgM		
Positive	24 (47.1)	14 (29.2)
Negative	16 (31.4)	24 (50.0)
Equivocal	2 (3.9)	0 (0.0)
Missing	9 (17.6)	10 (20.8)
Serotype (PCR)		
Den 1	0 (0.0)	0 (0.0)
Den 2	10 (19.6)	2 (4.2)
Den 3	5 (9.8)	2 (4.2)
Den 4	4 (7.8)	0 (0.0)
Other	1 (2.0)	0 (0.0)
Not detected	19 (37.3)	2 (4.2)
Not performed	12 (23.5)	42 (87.5)

Legend: Comparison of the percentage of various laboratory markers observed in dengue patients admitted to the ICU versus those in non-ICU wards. The data illustrated the differences in the prevalence of these markers between the two groups, providing insights into the laboratory profiles associated with different levels of disease severity.

## Data Availability

The data that support the findings of this study are available from the corresponding author, e.l.c.ong@ncl.ac.uk.

## Abbreviations

The following abbreviations are used in this manuscript:

DSS	Dengue Shock Syndrome
WHO	World Health Organization
HSAJB	Hospital Sultanah Aminah Johor Bahru
ICU	Intensive Care Unit
Non-ICU	Non-intensive Care Unit
BMI	Body Mass Index
Hb	Hemoglobin
Hct	Hematocrit
WCC	White cell count
CK	Creatinine kinase
LDH	Lactate dehydrogenase
ALT	Alanine aminotransferase
AST	Aspartate aminotransferase
ALP	Alkaline phosphatase
PT	Prothrombin time
APTT	Activated partial thromboplastin time
